# Supplementary material for: Malaria vaccine candidates displayed on novel virus-like particles are immunogenic and induce transmission-blocking activity
Source: PLoS One. 2019 Sep 10;14(9):e0221733. doi: 10.1371/journal.pone.0221733 (PMC6736250; doi:10.1371/journal.pone.0221733)
Supplement: S1 Table — (DOCX) [file pone.0221733.s002.docx]

**S1 Table Vaccine groups for Pfs230c-dS/dS VLP rabbit immunisations**

| **Rabbit ID** | **Total VLP protein (μg)** | **Total Pfs230c-dS protein (μg)** | **Alhydrogel**  **(with or without)** |
| --- | --- | --- | --- |
| R1864, R1865 | 20 | 6 | - |
| R1866, R1867 | 20 | 6 | + |
| R1868, R1869 | 100 | 30 | - |
| R1870, R1871 | 100 | 30 | + |

Note: Estimated incorporation of Pfs230c-dS into VLPs was ~30%, determined by Coomassie stained gels.
