# Supplementary material for: Malaria vaccine candidates displayed on novel virus-like particles are immunogenic and induce transmission-blocking activity
Source: PLoS One. 2019 Sep 10;14(9):e0221733. doi: 10.1371/journal.pone.0221733 (PMC6736250; doi:10.1371/journal.pone.0221733)
Supplement: S2 Table — (DOCX) [file pone.0221733.s003.docx]

**S2 Table Vaccine groups for Pfs230D1M-dS/dS VLP rabbit immunisations**

| **Rabbit ID** | **Total VLP protein (μg)** | **Total Pfs230D1M-dS protein (μg)** | **Adjuvant** |
| --- | --- | --- | --- |
| R1917, R1918 | 100 | 24 | Freund’s |
| R1919, R1920 | 100 | 24 | Alhydrogel |

Note: Estimated incorporation of Pfs230D1M-dS into VLPs was ~24%, determined by Coomassie stained gels.
