# Supplementary material for: Malaria vaccine candidates displayed on novel virus-like particles are immunogenic and induce transmission-blocking activity
Source: PLoS One. 2019 Sep 10;14(9):e0221733. doi: 10.1371/journal.pone.0221733 (PMC6736250; doi:10.1371/journal.pone.0221733)
Supplement: S3 Table — (DOCX) [file pone.0221733.s004.docx]

**S3 Table Vaccine groups for Pfs25-dS/dS VLP rabbit immunisations**

| **Rabbit ID** | **Total VLP protein (μg)** | **Total Pfs25-dS protein (μg)** | **Adjuvant** |
| --- | --- | --- | --- |
| R1825, R1826 | 100 | 3 | Freund’s |

Note: Estimated incorporation of Pfs25-dS into VLPs was ~3%, determined by Coomassie stained gels.
